# Epidemiology of Irritable Hip in Western Scotland: A Follow-Up Study

**DOI:** 10.7759/cureus.10036

**Published:** 2020-08-26

**Authors:** Ahmer Irfan, Anna Rose, Bryn Roberts, Steven Foster, James S Huntley

**Affiliations:** 1 Department of Surgery, Johns Hopkins Hospital, Baltimore, USA; 2 Department of Surgery, Queen Elizabeth University Hospital, Glasgow, GBR; 3 Critical Care, University Hospital Crosshouse, Kilmarnock, GBR; 4 Department of Paediatric Emergency Medicine, Royal Hospital for Children, Glasgow, GBR; 5 Department of Emergency Medicine, Royal Hospital for Sick Children, Glasgow, GBR; 6 Department of Surgery, Sidra Medicine, Ar-Rayyan, QAT

**Keywords:** irritable hip, transient synovitis, epidemiology and biostatistics

## Abstract

Background

A ‘limping child’ commonly presents to the emergency department (ED). In the absence of trauma, many are diagnosed with irritable hip (IH). The aetiology of IH is not well understood and there may be geographical and seasonal variations. We previously established one year (2016) epidemiological data of IH presenting to the Royal Hospital for Children (RHCG) ED in Glasgow, Scotland. The sentinel findings in that year were (i) an age distribution shift to younger (peak at two years of age), (ii) no marked association with social class, and (iii) a spring preponderance. We sought to strengthen or refute these findings by repeating our study to obtain comparative data for 2017.

Methods

We performed a retrospective analysis of all children discharged from the RHCG ED from January to December 2017. Relevant discharge codes were determined, and patient records screened. Patients without a discharge code had their presenting complaint and medical record screened. These data were compared to that of the previously published study from the same ED (2016).

Results

Several findings were consistent with the conclusions of the 2016 study. The incidence was similar with 362 and 354 cases diagnosed in 2017 and 2016 respectively. The boy-girl ratio was consistent across both data-sets, 2:1 and 1.9:1 respectively. The mean age of presentation was similar (3.3 vs 3.5 years) across both years, with the same medians (three years) and peaks (two years). There was no overt difference in incidence or correlation to social deprivation. However, in 2016, a spring preponderance was seen whereas there was an autumn preponderance in 2017. Pooling data from the two cohorts, 93% (n=668) of patients were managed exclusively by ED physicians, with 70% (n=504) not requiring any further follow-up. The majority of patients who required follow-up were seen in ED clinics (169/212, 79.7%). No patient initially diagnosed as IH was found to have septic arthritis (SA).

Conclusion

In this follow-up study, we again found (i) a younger age profile than other studies, and (ii) no overt association with social deprivation. The major difference between the previous (2016) and current (2017) study was the apparent seasonal peaks: spring (2016), and autumn (2017). This difference does not negate the 'antecedent infection' hypothesis, but any aetiological proposal should be capable of accounting for this discrepancy. Additionally, our studies highlight that the majority of these patients can be managed in the ED alone.

## Introduction

Irritable hip (IH) is a common, time-limited benign condition that is often seen in the paediatric emergency departments (ED) [1]. Its most common presenting symptom is a limp though patients can complain of pain, restricted range of motion and/or a low grade fever [2-4].

IH is classically seen as a diagnosis of exclusion, ensuring that more serious conditions such as septic arthritis (SA), osteomyelitis, bone tumours, leukaemia, Perthes disease, slipped capital femoral epiphysis (SCFE) and traumatic injury are ruled out [5]. However, after a sufficient history and examination, investigations in these patients can be selective and not all patients require a complete laboratory or radiological work-up [6].

Early childhood presentation (age between three and eight years) is usual [1]. Although many reports on this condition are European, there is some evidence that the disease is prevalent in other ethnicities [6]. The exact aetiology is not established, but the hypothesis of an antecedent viral infection, most commonly from the upper respiratory tract, is commonly suggested [2,7-9]. It has been argued that an infective or post-infective aetiology would lead to seasonal variation, though the epidemiological studies are not concordant [6,10-12].

We previously established one year (2016) epidemiological data pertaining to IH presenting to the Royal Hospital for Children Glasgow (RHCG) ED, Scotland (with IH defined as benign hip irritability without serious underlying pathology) [6]. The sentinel findings were (i) an age distribution shift to younger (peak at two years of age), (ii) no marked association with social class, and (iii) a spring preponderance. We sought to strengthen or refute these findings by repeating our study protocol for a further year (2017).

## Materials and methods

Hospital and locale 

The Glasgow 2011 Census documented the age and ethnicity of children in the Greater Glasgow and Clyde (GG&C) Health Board. This was used to determine the incidence of disease in the population. The total population of children aged 0-14 years in the GG&C area was 179,448. The RHCG is the only Children’s ED in the City of Glasgow (CoG) and the tertiary referral centre for the West of Scotland. This geographic restriction implies majority case-capture for children, although many patients with IH could still be treated or observed in primary care. An implicit assumption is made that the population of people aged 0-14 years has remained static from the time of census to present.

Retrospective analysis and search methodology 

Data were collected (work phase: April to November 2019) concerning all patients presenting to the RHCG ED over 12 months: January 1 to December 31, 2017. On discharge from the ED at initial visit, patient episodes were coded on TrakCare® (InterSystems TrakCare Lab, Cambridge, MA) based on their ED diagnosis. Notes for all potentially relevant discharge codes were screened (n=421) to determine diagnosis if available. Within this group, 396 patients had a discharge diagnosis of transient synovitis (TS)/IH and 25 patients had other discharge diagnoses. The presenting complaint and records of all patients who did not have an ED discharge code were also screened during this time frame to ensure no cases were missed (n=6054). This data collection method was similar to the earlier study for IH cases in 2016 [6].

Inclusion/exclusion criteria 

Children were included from birth to the age of 14 years at the time of presentation. All patients who were diagnosed with IH based on clinical examination, investigations, or both were included. They were excluded if an alternative diagnosis was determined during follow-up, after their ED discharge. Patients with a preceding history of accidental or non-accidental trauma were also excluded. Patients with multiple presentations during the same episode (within 14 days of initial presentation) were screened and excluded with only the initial presentation included in the analysis.

Social deprivation 

Social Deprivation was assessed using the Scottish Index of Multiple Deprivation 2016 (SIMD). The SIMD is a national deprivation score allocated by the Scottish government ranking areas from 1 (most deprived) to 6976 (least deprived). Each area was divided to contain similar population sizes (760 people per area). Deprivation was determined using 38 markers, grouped into seven domains which considered (1) Income, (2) Employment, (3) Education, (4) Health, (5) Access to Services, (6) Crime, and (7) Housing. The total population and working population for all of the ranking areas in the CoG was obtained to determine incidence.

Seasonality

Season of presentation was determined using the Northern Meteorological Definition [13] which grouped the seasons into spring (March to May), summer (June to August), autumn (September to November), and winter (December to February).

Statistical analysis 

Where appropriate, data were analysed for statistical significance using Stata 15.0 (StataCorp, College Station, TX).

## Results

Diagnosis of IH

IH was diagnosed in 362 patients over the one-year study period (January-December, 2017), comparable to the previous study (2016): 354 patients.

In our previous study, all patients discharged with a diagnosis of IH had arranged follow-up, the majority of these patients (n=197, 55.6%) did not attend and many of those who did (n=133, 37.6%) reported resolution of symptoms [6]. Therefore, the ED changed their policy for these patients to limit wasted appointments. Parents of patients discharged with IH were given a telephone number to make an appointment if symptoms had not resolved within seven days of onset. With this method, 218 patients (60.2%) were discharged and did not require ED follow-up. Of those who made an appointment (n=136), 25.7% (n=35) were seen in an ED clinic. Unfortunately, the majority (n=101, 74.3%) of patients did not attend their appointment. A minority of patients seen for IH were coded as being admitted (n=8, 2.2%). All these patients were moved from the ED to the Clinical Decision Unit (CDU), which is a short stay observation area for patients requiring a longer stay for further investigations or assessment by a consulting speciality. The majority (n=6) of these patients were subsequently discharged from the CDU. Of the two patients admitted to the inpatient floor, one was admitted as their presentation was late at night (and was subsequently discharged in the morning), the other was admitted to the orthopaedic floor for monitoring following a fall. In 22 cases (6.1%), a sub-speciality (orthopaedics, n=21; rheumatology, n=1) was consulted for further diagnostic assessment or outpatient follow-up on the initial presentation.

Geographical restrictions

The Glasgow 2011 census provided population data for the GG&C health board and SIMD provided population data within the CoG council area. The postcode of each patient was screened, if they were outside the respective health board or council area, they were removed from the incidence analysis. From the 2017 study population of 354 children, the number of children in the GG&C and CoG areas were 326 and 202, respectively. The GG&C population was used for analysis of overall incidence and variations due to age and sex. The CoG population was used for analysis of incidence variation by social deprivation status.

Patients

The annual incidence of IH in GG&C (n=326) was 181.7 per 100,000 children. This was similar to 177.7 per 100,000 the previous year. There was a greater incidence of IH in boys, with a 2:1 boy:girl ratio (217 boys, 109 girls p<0.01), again comparable to the 1.9:1 ratio in 2016. There was no overt difference in the laterality of the hips in either year, though a slight variation in the side that presented most commonly (51.7% right-sided in 2017, 51.9% left-sided in 2016).

Alternative diagnoses

Of the patients initially coded as TS/IH (n=396), 34 were believed to have a different final diagnosis. These were TS of a different joint (n=8), Perthes disease (n=9), fracture (n=4), soft tissue injury (n=2), reactive arthritis (n=1), osteomyelitis (n=1), systemic lupus erythematosus (n=1), lymphatic haemorrhage (n=1), juvenile idiopathic arthritis (n=1), external snapping hip (n=1), discitis (n=1) and hypermobility (n=1). In three patients, no final diagnosis was documented. These results are similar to the prior study [6] and Table 1 demonstrates the pooled data of the most common alternative diagnoses. No cases of SA were mistakenly coded as IH.

A subset of patients (n=9) across the two years were initially thought to have TS, however, their symptoms were ultimately attributed to fracture pathology. Injury patterns seen were Toddler's fracture (n=3), distal tibial fractures (n=2), and greater trochanter fracture (n=1). The final three patients had symptoms attributed to previous fractures that had now healed (two tibial and one fibular).

**Table 1:** Most common alternative diagnoses for patients initially coded as irritable hip (IH) in all patients presenting between January 2016 and December 2017 (n=804)

Alternative Diagnosis	Number of Patients
Transient Synovitis of alternative joint	20
Perthes Disease	11
Fracture	9
Soft Tissue Injury	8
Juvenile Idiopathic Arthritis	4
Diagnosis not known	12

Seasonality

**Figure 1:**
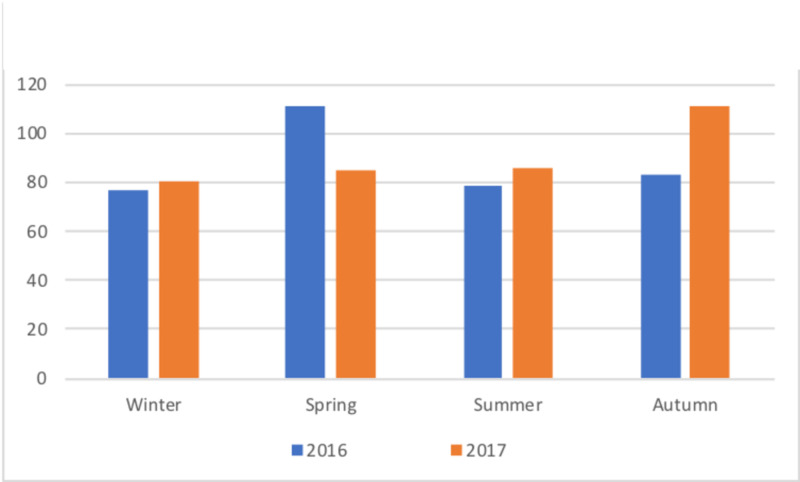
Season of presentation

**Figure 2:**
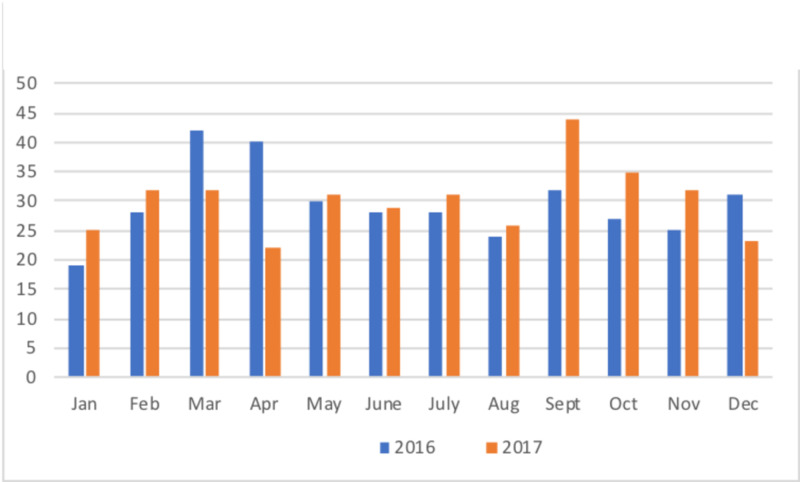
Month of presentation

In 2016, there was a slight spring preponderance (n=111, 31.3%) [6]. This was not present in 2017, showing a smaller proportion of patients presenting in spring (n=85, 23.5%), and an autumnpreponderance (n=111, 30.7%) (Figure 1). When analysed by month, the major changes were a decrease in numbers for March (32 vs 42) and April (22 vs 40), with a corresponding increase in September (44 vs 32) (Figure 2).

Age at presentation

**Figure 3:**
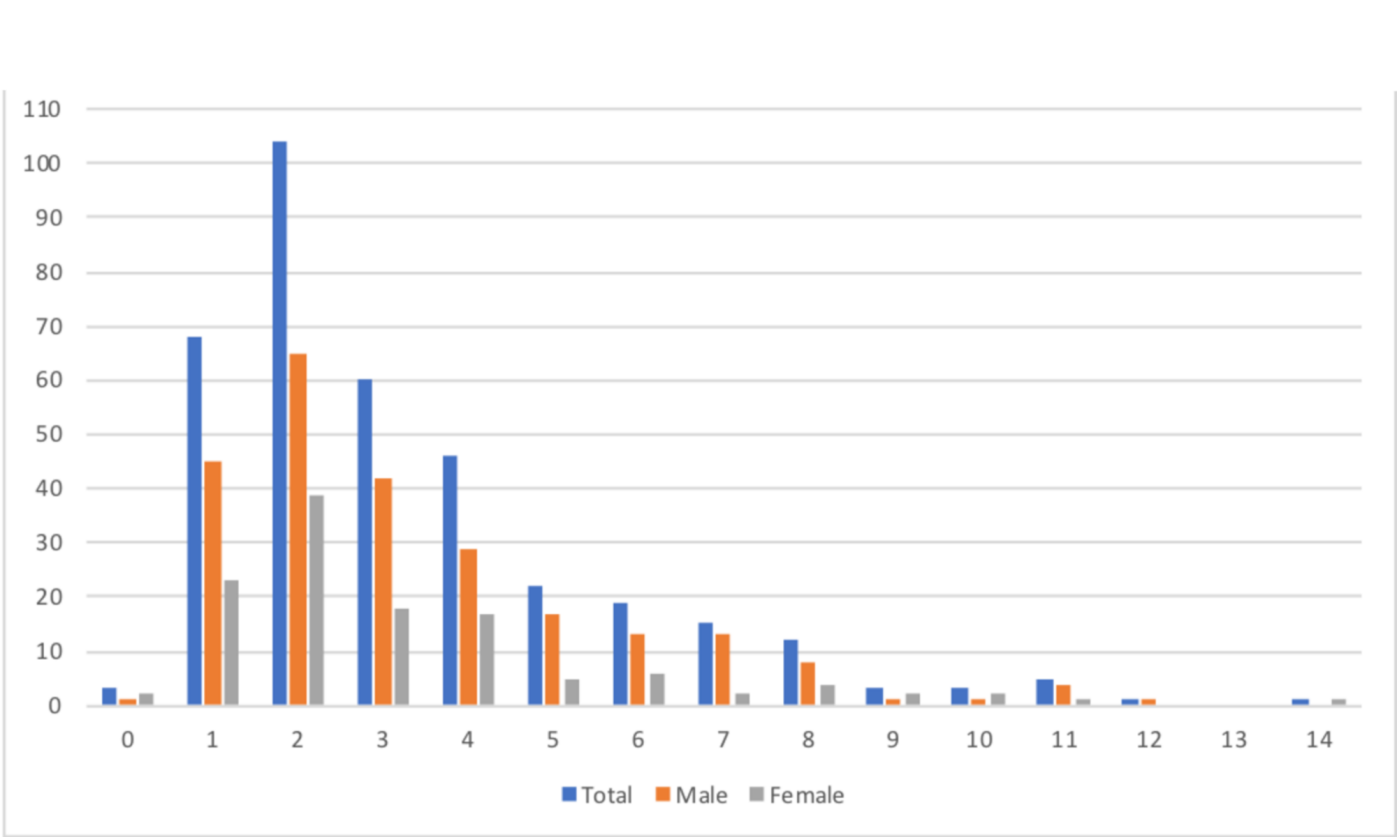
Age and Incidence in 2017

A surprising finding in 2016 was the shift in age distribution to younger (mean 3.5 years) compared to that in analogous studies [6]. In 2017, there was a similar shift, with mean, median, and mode of 3.3, three, and two years, respectively (Figure 3).

Social deprivation

The SIMD divided Glasgow into 743 areas; they were ranked as per the SIMD domains. Due to the geographical variation of the patient population, a smaller proportion (n=202) were included in this analysis. There is a slight increase in the proportion of patients in the 2017 cohort from the CoG area (55.8% vs 53.3%) [6].

A substantial proportion of these data zones were ranked in the 1st quintile (most deprived), containing significantly more children. However, there was no variation in incidence or trend based on SIMD quintile (Table 2). This is analogous to the 2016 study and similarly when the data are combined. There are slight differences in the incidence in particular quintiles between the two years.

**Table 2:** Social deprivation and incidence of disease SIMD: Scottish Index of Multiple Deprivation

SIMD quintile	Number of zones	Non – working age population	Number of patients (2016)	Number of patients (2017)	Incidence (2016)	Incidence (2017)	Incidence (Combined)
1	358	94725	100	100	105.6	105.6	105.6
2	125	29806	34	40	114.1	134.2	124.1
3	106	23335	24	20	102.8	85.7	94.3
4	83	17866	15	23	84.0	128.7	106.3
5	71	16595	16	19	96.4	114.5	105.5

## Discussion

The incidence of IH in Glasgow has remained consistent between the two years (181.7 vs 177.7 cases per 100,000 children) [6] and is comparable to studies from Sweden (203 cases per 100,000 children) [11] and Edinburgh (142 cases per 100,000 children) [2].

The younger age of presentation of IH (mean 3.5 years in 2016) [6] was replicated in 2017 (mean age 3.3 years) with a peak at two years of age. Typically IH is reported elsewhere in the literature as occurring between three and nine years [1], with a mean of six years [8,11]. It has been previously hypothesised that an infective or post-infective intra-articular process may underly IH, which is generally thought to be rare under three years of age. A commonly associated pathogen with the younger age group is Kingella kingae, usually presenting with less severe symptoms than other infections and may be easily missed [14]. To fully explore such an association would require blood and/or synovial fluid cultures. Although it may be possible to justify further testing, the majority of children presenting with IH at our institution had complete resolution of symptoms.

The aetiology of IH is still debated [12], though the ‘antecedent infection’ theory, usually from the upper respiratory tract, is commonly favoured [2,7-9], with some evidence of elevated viral titres, though no specific infectious agent identified [15]. Based on this hypothesis, there might be an expectation that an increase would occur when viral illnesses, in general, are most prevalent (autumn and winter); this theoretical association has not been substantiated. Our 2017 data shows an autumn preponderance, consistent with a Scandinavian study showing increased cases in October/November [11]. However, this is refuted by our 2016 data, showing a spring preponderance [6]. A large epidemiological study from Liverpool, UK, did not exhibit seasonality [10]. A driver for obtaining further epidemiological data 2016-7 was that the excess pattern found in a particular single year need not necessarily indicate a correlation with seasonality per se [16]. Indeed, we have found this to be the case: across the two years, there is no correlation of incidence with season or months. Although long term (10- or 20-year) data would provide a stronger conclusion, current evidence does not support a seasonal link in our population.

An association between Perthes disease and social deprivation has been proposed [17,18] and also disputed - specifically in Glasgow [19] and South West Scotland [20]. A link to social deprivation has also been proposed for IH in Liverpool [10]. A social class association was not demonstratable for IH in Glasgow in either 2016 [6] or 2017. In both years, the highest incidence was in quintile 2, with similar incidences in quintiles 1, 4, and 5 when the data is combined. We suggest that social deprivation variability within Glasgow does not have a marked effect on the risk of IH.

IH is purportedly a diagnosis of exclusion because it is important not to miss differential diagnoses that require urgent intervention, of which SA is the most feared. Across both years, it was encouraging that no patient with SA was misdiagnosed with IH [6]. TS of other joints was included initially with the IH population, as they share the same code in our electronic healthcare system. This is a clear limitation to accurately tracking IH, and we have proposed modification of the system to enhance granularity i.e. distinguish between IH and irritable other joint. Excluding these, the proportion of patients who were initially coded as IH and subsequently found to have a different diagnosis was similar across the two years (7.8% in 2016 vs 6.6% in 2017). Although there is room for improvement in our diagnosis and exclusion of other conditions, our data demonstrates the approach taken by our ED has not led to patient harm.

In our initial study, IH was shown to be a frequent presentation to the paediatric ED, with physician time often allocated for follow-up that was not attended. The average clinic appointment is scheduled for 10 minutes; 197 patients not attending equates to 33 hours of appointment time unfilled [6]. By changing the system to an ‘opt-in’ system, the total number of appointments between the two years was greatly reduced (354 vs 136). Unfortunately, there was an even greater percentage of ‘did not attend (DNAs)’ in 2017 (74.3% vs 55.6%), for no clear reason. Despite this, the amount of time lost due to DNAs almost halved (to 17 hours), an improved utilisation of health resources. Following initial ED assessment, a minority of patients required admission to the inpatient unit (n=2) or referral to another speciality for further assessment or outpatient follow up (n=22). This further shows that such patients can generally be successfully managed by the ED team.

## Conclusions

In this follow-up study, we found redemonstration of (i) a younger age profile than other studies, and (ii) no association with social deprivation. The major difference between the two cohorts concerned the apparent seasonal peaks: spring for 2016, and autumn for 2017. This difference does not of itself negate the 'antecedent infection' hypothesis, but any aetiological proposal should be capable of accounting for this discrepancy. Additionally, our studies seem to highlight that the majority of these patients can be managed appropriately by the paediatric ED, without requiring onward speciality referral. For these patients, we also propose an ‘opt-in’ method of follow-up.
